# Long noncoding RNAs involved in therapeutic response: implications for cervical cancer drug resistance

**DOI:** 10.3389/fonc.2025.1644104

**Published:** 2025-10-21

**Authors:** Samuel Trujano-Camacho, Carlos Contreras-Romero, Verónica García-Castillo, David Sánchez-Marín, Mercedes Olvera-Valencia, Mauricio Rodríguez-Dorantes, Oscar Peralta-Zaragoza, David Cantú de León, Eduardo López-Urrutia, Carlos Pérez-Plasencia

**Affiliations:** ^1^ Laboratorio de Genómica, Unidad de Biomedicina, Facultad de Estudios Superiores-Iztacala (FES-IZTACALA), Universidad Nacional Autónoma de México (UNAM), Tlalnepantla, Mexico; ^2^ Laboratorio de Diagnóstico Molecular Genelab, Cuahutémoc, Mexico; ^3^ Posgrado en Ciencias Biológicas, Facultad de Medicina, Universidad Nacional Autónoma de México (UNAM), Coyoacan, Mexico; ^4^ Laboratorio de Virus y Cáncer, Dirección de Investigación, Instituto Nacional de Cancerología, México City, Mexico; ^5^ Laboratorio de Oncogenómica, Instituto Nacional de Medicina Genómica, Tlalpan, Mexico; ^6^ Dirección de Infecciones Crónicas y Cáncer, Centro de Investigación Sobre Enfermedades Infecciosas, Instituto Nacional de Salud Pública, Cuernavaca, Mexico; ^7^ Dirección de Investigación, Instituto Nacional de Cancerología, México City, Mexico

**Keywords:** lncRNAs, non-coding RNAs, chemoresistance, cancer, cervical cancer

## Abstract

Cervical cancer (CC) remains among the top causes of death for women worldwide, especially in low-income countries, where screening strategies are less widespread. Treatment strategies are mainly based on DNA-damaging agents, though resistance mechanisms still pose a substantial challenge. Among the cellular components that mediate treatment resistance, long non-coding RNAs (lncRNAs) stand out because of their broad regulatory effects. They are involved in virtually all drug resistance mechanisms, such as drug efflux, DNA repair, evasion of cell death, and aberrant epigenetic modifications. Although resistance mechanisms are fundamentally similar in most cancers, the underlying regulatory networks vary substantially. Here, we review the literature for lncRNAs involved in treatment resistance mechanisms in general, and then focus on lncRNAs that mediate resistance in CC. We found a broad area of opportunity in lncRNA research in resistant CC, as the lncRNAs involved are still to be described. These master regulators are promising candidates for response markers and therapeutic targets. May this compilation serve as the basis for further descriptions of the regulatory roles of lncRNA in CC treatment resistance.

## 1 Introduction

Cervical cancer (CC) is the fourth most common cancer among women worldwide and the third leading cause of cancer-related mortality. In 2022 alone, 662,044 cases and 348,709 deaths from cervical cancer occurred, with mortality exceeding 50% in many cases. While most cases are detected in high-income countries, the number of deaths is disproportionately high in low-income countries, where CC ranks as the second most deadly cancer for women (1).

Human Papillomavirus (HPV) infection is accepted as the main etiology of CC. HPV virions can reach any of the different epithelial regions that comprise the uterine cervix: the endocervix, lined by columnar glandular epithelium, the ectocervix, lined by stratified squamous epithelium, or the transformation zone formed by metaplastic epithelium (2), giving rise to two histological variants: squamous cell carcinoma (85%) and adenocarcinoma (15%) (3).

Current pharmacological treatment strategies for CC include platinum-based chemoradiation therapy (CCRT) and brachytherapy. For advanced or recurrent cases, a platinum-based chemotherapy doublet, often combining cisplatin with paclitaxel, ifosfamide, gemcitabine, topotecan, or vinorelbine, to mention a few, is the first-line treatment. However, the response rate for early-to-intermediate stages (IIA-IIIB) is only 68%, and 5-year overall survival (OS) and progression-free survival (PFS) rates remain low, at 68% and 66%, respectively (4). Several groups are currently working to optimize these effects through novel drug delivery strategies of already approved drugs. These strategies include delivery of the recently approved pembrolizumab through nanoparticles of nanopolymers (5). Nonetheless, resistance to therapy –whether intrinsic or acquired– remains a critical challenge, undermining treatment efficacy and contributing to progressive, advanced, or metastatic disease.

Therapy resistance in CC, as in other cancers, arises through molecular mechanisms involving genetic and epigenetic alterations. Cancer stem cells (CSCs) play a prominent role, alongside mechanisms such as enhanced drug efflux, increased DNA repair, evasion of apoptosis, autophagy, epithelial-mesenchymal transition (EMT), and aberrant epigenetic modifications (6).

Recent research highlights the involvement of long noncoding RNAs (lncRNAs) in these processes, positioning them as pivotal players in therapy resistance. LncRNAs are RNA molecules longer than 200 bp that, while not typically translated into proteins, fold into complex secondary structures, enabling interactions with DNA, RNA, or proteins. These interactions allow lncRNAs to act as molecular decoys, signaling mediators, guides, and scaffolds for protein complexes (7). Deregulated lncRNAs have been shown to influence every hallmark of cancer, promoting malignant transformation and tumor progression. Multi-omics studies have further demonstrated that lncRNAs contribute directly to a cancerous phenotype by interacting with various macromolecules and molecular complexes (8).

**Figure 1:**
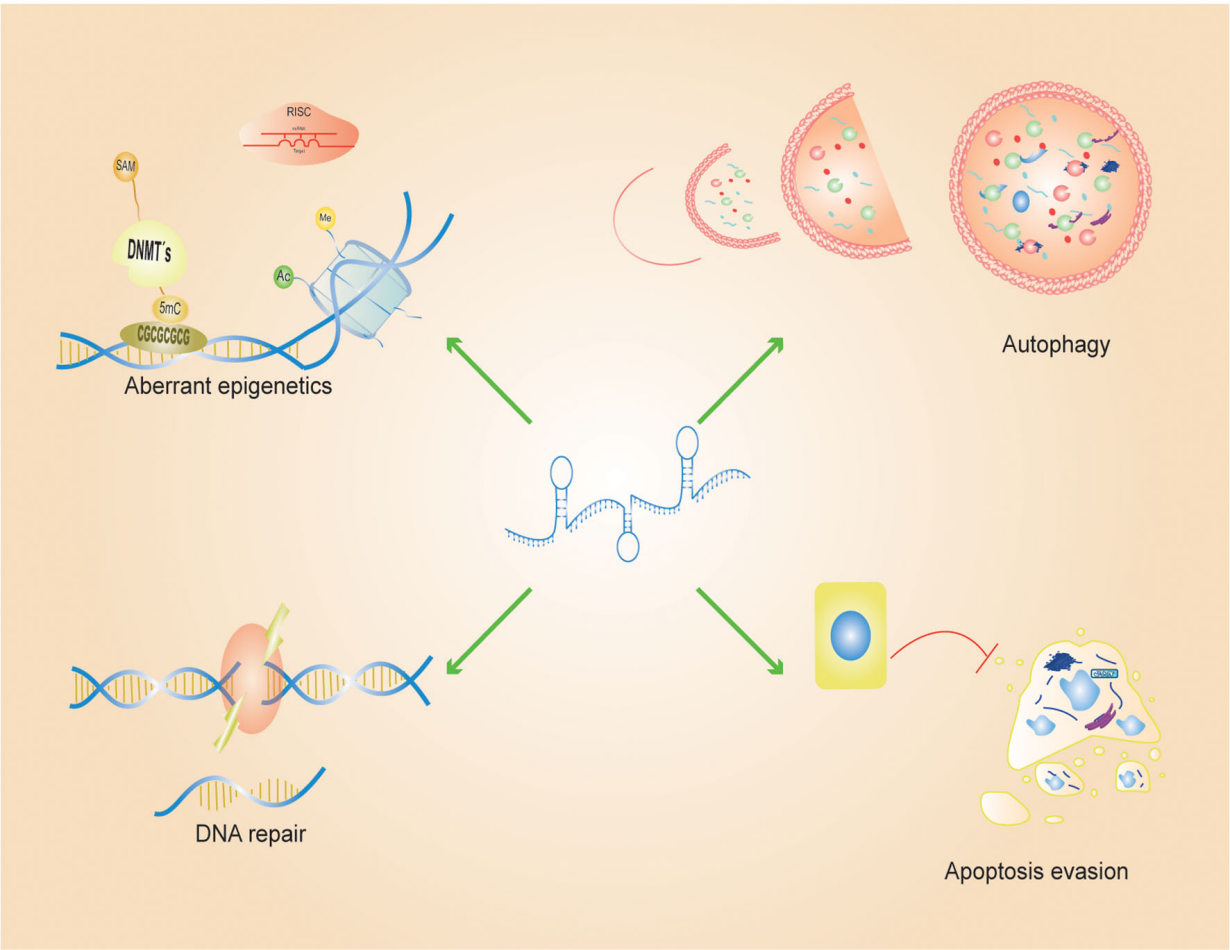
LncRNA-mediated resistance processes: Aberrant epigenetic processes, autophagy, DNA repair, evasion of apoptosis and epithelial-mesenchymal transition are some of the main processes mediated by LncRNAs in cancer. To promote a chemoresistant phenotype, long non-coding RNAs (LncRNAs) modulate gene expression through diverse epigenetic mechanisms, including DNA methylation, histone modifications, and functioning as competing endogenous RNAs (ceRNAs) that sequester microRNAs (miRNAs). Beyond epigenetic regulation, LncRNAs bolster cellular resistance to therapy-induced cytotoxicity by activating autophagy which enhance tumor cell survival and metastatic potential. Furthermore, LncRNAs play a pivotal role in preserving genomic stability by facilitating the repair of single- and double-strand DNA breaks and by interfering with apoptotic signaling pathways, thereby enabling cancer cells to evade programmed cell death and sustain malignant progression.

This review focuses on the role of lncRNAs in mediating drug resistance in CC. First, we explore their involvement in known resistance mechanisms such as drug efflux, DNA repair, and epigenetic modifications. Then, we analyze reported associations between specific treatments and lncRNAs, emphasizing their potential as biomarkers for predicting therapeutic response and guiding treatment strategies. By understanding their molecular and clinical significance, we aim to underscore the promise of lncRNAs as targets to overcome resistance and improve outcomes for CC patients. Many of these lncRNAs affecting resistance mechanisms have yet to be characterized in the context of cervical cancer, highlighting the need for further research and raising important new questions for future investigation in this field (**Figure 1**).

## 2 lncRNAs involved in resistance mechanisms

The sensitivity of each tumor to different chemotherapy drugs and the determinants of their specificity depend on various factors, including the tumor microenvironment and dynamic changes that activate specific mechanisms, such as those mentioned above (9). In this regard, several studies have described the involvement of lncRNAs in resistance mechanisms. Although those reviewed below were not necessarily performed in cervical cancer (CC), they demonstrate that long noncoding RNAs are implicated in drug resistance and sensitivity in cancer cells (10). Understanding how lncRNAs modulate drug response is the basis for new avenues to address resistance to anticancer therapies.

### 2.1 Reduced absorption and increased drug efflux

One of the main resistance mechanisms is reduced drug uptake and increased drug efflux, which are primarily mediated through an increase in the ATP-binding cassette (ABC) superfamily transporters (11). There are 48 known transporters, classified into seven subfamilies (ABC A to G); 30 of them contribute to the development of drug resistance in tumors, most notably P-glycoprotein (P-gp) and breast cancer resistance protein (BCRP/ABCG2) (12). Several lncRNAs play crucial roles in regulating these drug transporters.

While much of the evidence comes from studies in other cancer types, these findings provide valuable insights for cervical cancer (CC). For example, the lncRNA HOTAIR has been studied in gastric cancer, where its overexpression regulates the activity of P-glycoprotein (P-gp), promoting the efflux of drugs such as doxorubicin and paclitaxel (13). MALAT1 plays a significant role in lung and gastric cancer, increasing resistance to cisplatin. Its ability to modulate ABC transporters and signaling pathways suggests a direct impact on treatment efficacy (14). Considering that both lncRNAs are overexpressed and associated with a poor prognosis in CC (15, 16) and that they promote resistance to drugs used in CC treatment regimens, we can begin to explore the implications of lncRNAs in specific tumors and as resistance mechanisms shared among tumors dependent on the presence of specific regulators, such as MALAT1 and HOTAIR. Similarly, reviewing other lncRNAs involved in similar resistance mechanisms can potentially lead us to new, unexplored CC targets. The lncRNA MRUL (multidrug resistance-related and upregulated lncRNA), for example, regulates ABCB1 expression in gastric cancer, thereby promoting multidrug resistance (17). These findings highlight the direct role of specific lncRNAs in modulating ATP-binding cassette (ABC) transporters and driving the multidrug-resistant phenotype in various cancer types.

### 2.2 Increased DNA repair and apoptosis evasion

Various genotoxic treatments damage DNA by breaking covalent bonds between nucleotides, disrupting genome replication and transcription. The repair machinery can intervene by correcting the damage at specific checkpoints through a signaling cascade or by triggering cell death (18). The DNA damage response induces apoptosis through the so-called intrinsic pathway, characterized by membrane permeabilization and release of pro-apoptotic factors such as cytochrome C or apoptotic protease activation factor 1 (Apaf-1) into the cytosol. Resistance mechanisms usually involve deregulation of proteins involved in the intrinsic apoptosis pathway, including members of the Bcl-2 family and the p53 tumor suppressor, overexpression of inhibitors of apoptosis proteins (IAPs), or overactivation of signaling pathways such as PI3K/AKT, even in the presence of DNA damage (19).

LncRNAs play a key role in DNA damage repair and apoptosis avoidance through regulating the ATM, ATR, and p53 pathways (20). Homologous recombination (HR) stands out among the DNA repair mechanisms and is the most studied in cancer. It is regulated by ATM, ATR, BRCA1-2, and RAF5, to name a few (21). The lncRNAs NEAT1, ANRIL, and ScaRNA2 perform regulatory roles in HR by stabilizing ATM and ATR, thus increasing the rate of DNA repair and conferring resistance to cancer cells (22, 23).

In this context, NEAT1 and ANRIL are markedly overexpressed in cervical cancer tissues and cell lines, correlating with more severe clinical features and reduced patient survival (24–26). Suppressing NEAT1 expression hampers cervical cancer glycolytic activity, which has been widely associated with high PD-L1 expression, as well as with the inhibition of apoptosis, lactate production, and immunosuppressive processes (27), whereas its upregulation produces the opposite effect. NEAT1 contributes to cervical cancer progression through multiple pathways, including the NEAT1/miR-133a/SOX4 axis, and the WNT/β-catenin/PDK1 signaling cascade axis (28, 29) both pathways are widely associated with the evasion of apoptosis, which is achieved through the transcriptional activation of genes such as survivin (30, 31). Meanwhile, ANRIL inhibition reduces the apoptosis evasion capabilities of CC cells (32).

Similarly, lncRNAs such as APAF1-binding lncRNA (ABL) or LINC00942 have been implicated in drug resistance processes by promoting the escape from apoptosis. ABL competes directly with cytochrome C to interact with apoptotic protease-activating factor 1 (APAF1), thereby preventing apoptosome formation (33). LINC00942, in turn, regulates c-Myc mRNA stabilization through the LINC00942/MSI2/c-Myc axis, preventing apoptosis as well (34).

### 2.3 Autophagy

Autophagy is a normal cell death process; however, cancer cells resort to autophagy to draw energy from macromolecules, thereby favoring growth and proliferation. Cervical cancer cell lines have shown resistance to drugs such as paclitaxel and cisplatin when autophagy is active, while autophagy-inhibiting drugs such as 3-methyladenine or chloroquine can restore drug cytotoxicity (35). Paclitaxel resistance is achieved through autophagy by activating HIF1α and the Warburg effect, while HIF2a promotes cisplatin drug resistance (36, 37). In addition, autophagy related to drug resistance is induced by elements and pathways dysregulated in cervical cancer, including PI3K/Akt/mTOR pathway, Beclin1, Bcl-2, Ras, p53, DUSP1, heparinase, HMGB1, and noncoding RNAs (38).

Along these lines, lncRNAs, such as LincRNA-p21 and HOTAIR, have positive feedback mechanisms that activate HIF1a transcription in CC (39, 40). This suggests that they could play an important role in resistance processes mediated by autophagy, though this has not yet been fully described.

Similarly, other lncRNAs, such as PVT1 in pancreatic cancer and EIF3J-DT and CRNDE in gastric cancer, have been associated with autophagy processes and resistance to gemcitabine and 5-fluorouracil (5-FU), respectively. Their high expression in CC could lead us to resistance mechanisms that have been little explored in this type of cancer (41–43).

### 2.4 Aberrant epigenetics

Given that chromatin structures strongly influence transcriptional activity, epigenetic events play a fundamental role, not only in tumor development and progression, but also in the generation of resistance to antitumor drugs (44). The cancer epigenome is characterized by changes in DNA methylation and histone modification, which allow tumoral progression (45). Several genes are hypomethylated and hypermethylated in cervical cancer, such as RASSF1A, TFPI2, MEG3, KLF4, IFN-γ, RAD51L3, and XRCC2 (46). Changes in DNA methylation lead to increased drug resistance through the upregulation of the PI3K/AKT, Wnt/β-catenin, Notch, and NF-κB signaling pathways. For instance, in cervical cancer, methylation of the Casp8A2 gene is sufficient to increase drug resistance; on the contrary, hypermethylation of WRN confers cervical cancer cells high sensitivity to the topoisomerase I inhibitor (CPT-11) (47).

The balance between histone deacetylases (HDACs) and histone acetyltransferases (HATs) also regulates gene transcription. For instance, in cervical cancer, MOF, a HAT enzyme, inhibits replication stress induced by cisplatin (48), while HDAC6 is involved in cell proliferation and motility (49).

HOTAIR is one such lncRNA; it may function as a protein scaffold for the Polycomb Repressive Complex 2 (PRC2), which modifies histone residues and binds DNMT3b to methylate promoter regions (50). On the other hand, lncRNA H19 modulates the activity of ALDH and activates the WNT/β-catenin pathway by interacting with miR-141, causing chemoresistance in colorectal tumors (51). H19 also interacts with let-7, upregulating LAT2 and OLR1 and leading to increased GSTM3TV2 levels in gemcitabine-resistant pancreatic tumor tissues (52). In ovarian cancer, NEAT1 knockdown releases miR-770-5p from sponging, which decreases PARP expression. Elevated PARP levels synergize with platinum compounds, improving the efficiency of chemotherapeutic approaches (53).

## 3 Long noncoding RNAs associated with therapy response

Only a handful of studies have associated lncRNAs with drug resistance in cervical cancer (CC), making it challenging to elucidate the mechanisms underlying drug resistance (54). Considering the therapeutic schemes (55) and the reported roles of lncRNAs in response to them, it is important to note that the expression profiles of lncRNAs promoting chemoresistance is similar between cervical cancer (CC) and other gynecological cancers. This allows us to gain insight on the possible role of lncRNAs that have not yet been described in CC, as the involved mechanisms are likely similar. This in-depth analysis could lead to new proposals for establishing markers based on the relationship between lncRNA expression levels and their involvement in drug-dependent resistance. (**Table 1**).

**Table 1:** LncRNAs are involved chemoresistance in CC.

Therapeutic line	Drug	LncRNA	Expression in CC	Source	Reference
First line	Cisplatin	H19	Down-regulated	SW480, HCT116 cell lines	(49)
		DANCR	Up-regulated	HeLa, C33 cell lines	(63)
		PVT1	Up-regulated	SiHa cell line	(65)
		ZFAS1	Up-regulated	HeLa, CaSki cell lines	(66)
	Carboplatin	SNHG12	Up-regulated	HeLa, SiHa cell lines	(70)
	Paclitaxel	lncRNA KB-1471A8.2	–	A2780, SKOV3 cell lines	(72)
		HEIH	–	Ishikawa, HHUA cell lines	(74)
		NEAT1	Up-regulated	hESC, Ishikawa, HHUA cell lines	(75)
		MALAT1	Up-regulated	DLBCL cell line	(77)
	PD-1/PD-L1 inhibitors	LINC00473	Up-regulated	SW‐1990 cell line	(78)
		UCA1	Up-regulated	SW480, SW620 cell lines	(93)
Second line	5-Fluorouracil	CCAT1	Up-regulated	HCT116, HT29 cell lines	(94)
	Bevacizumab	CRART16	–	SGC, KATO cell lines	(96)
		SNHG11	–	Colorectal cancer tissues	(97)
	Gemcitabine	HCP5	Up-regulated	Pancreatic cancer tissues, PANC-1, SW 1990 cell lines	(79)
		LINC00346	–	PANC-1, Capan-1 cell lines	(80)
	Paclitaxel	lncRNA KB-1471A8.2	–	A2780, SKOV3 cell lines	(72)
		HEIH	–	Ishikawa, HHUA cell lines	(74)
		NEAT1	Up-regulated	hESC, Ishikawa, HHUA cell lines	(75)
		MALAT1	Up-regulated	DLBCL cell line	(77)
	Irinotecan	HOTAIR	Up-regulated	Colorectal and Cervical cancer tissues	(84, 87)
		MALAT1	Up-regulated	Colorectal cancer tissues	(84)
		UCA1	Up-regulated	Colorectal cancer tissues	(85)
		H19	Up-regulated	Colorectal cancer tissues	(85)
		CRNDE	Up-regulated	Colorectal cancer tissues	(85)
Other recommended regimens	Gefitinib	CASC9	Down-regulated	PC9 cell line	(99)
		MITA1	–	HCC827, HCC827GR cell lines	(98)
		UCA1	Up-regulated	NSCLC tissues, PC9 cell line	(100)
		HOST2	Up-regulated	PC9 cell line	(101)
	Lapatinib	GIHCG	Up-regulated	gastric cancer, melanoma, hepatocarcinoma, thyroid cancer, breast cancer, pancreatic cancer, colorectal cancer cell lines	(104)
		SPINT1-AS1	–	gastric cancer, melanoma, hepatocarcinoma, thyroid cancer, breast cancer, pancreatic cancer, colorectal cancer cell lines	(104)
	Sunitinib	HOTAIR	Up-regulated	786-O, ACHN cell lines	(105)
		lncARSR	–	Renal cancer tissues, 786-O, ACHN cell lines	(106)

### 3.1 First-line therapy

The first treatments for CC patients are radiosensitizers, mainly platinum compounds such as Cisplatin and Carboplatin; these are often used in combination with microtubule polymerization inhibitors like Paclitaxel or topoisomerase-1 inhibitors such as Topotecan (55). Though in many cases, patient therapies must be reformulated due to the development of mechanisms that promote or enable resistance to drugs and first-line therapies (56) (**Table 1**).

The main mechanisms by which tumor cells become resistant to first-line platinum drugs are drug efflux, DNA repair, and evasion of cell death (57). An enhanced DNA damage response bypasses the effect of cisplatin therapy in several ways. For instance, the heterodimer ERCC1-ERCC4 removes DNA bulky lesions through Nucleotide Excision Repair (NER) (58). Thus, ERCC1 is negatively correlated with responsiveness to cisplatin, which makes it a prognostic factor (59). The DNA mismatch repair pathway is also implicated, through the overexpression of MSH2 and downregulation of PMS2 (60, 61).

Furthermore, cisplatin induces apoptosis to achieve its antitumoral effect; however, in CC cells, the mechanisms leading to apoptosis are dysregulated, and cancer cells escape death. This dysregulation includes overexpression of antiapoptotic proteins such as BCL-2, Bcl-XL, and Bag-1; and downregulation of caspase activators, which decreases caspases in patients with drug resistance (62). Cisplatin-induced apoptosis requires an active MAPK, while deactivation of other pathways due to mutation or loss of P53 and NK-κβ reduces chemotherapy efficacy (60, 63, 64).

#### 3.1.1 Cisplatin

It has been proven that some lncRNAs are directly implicated in cisplatin resistance in CC. For example, DANCR binds to miR-665, increasing the nuclear distribution of SMAD by overexpression of TGFBR1 (65). GAS5 sponges miR-21, enhancing the expression of phosphorylated STAT3 and E2F3; this decreases TIMP3 and PDCD4, leading to a tumoral cisplatin-resistant phenotype (66). PVT1 overexpression is associated with poor prognosis. It binds to and stabilizes MYC and Nuclein, contributing to cervical cancer development and its inhibition has also been strongly associated with sensitivity to cisplatin treatment (67). ZFAS1 has been reported to be upregulated in CC tissues. Its increased expression indicates poor prognosis, and its silencing enhances cisplatin sensitivity; however, the mechanism by which this effect occurs is unknown (68).

Several studies point to UCA1 promoting cisplatin resistance in cervical cancer-derived cells through regulation of caspase-3, p21, CDK and survivin expression (69). Recently another mechanism was demonstrated in which UCA1 acts as a sponge for miR-195-5p, targeting IKBKB promoting cisplatin resistance (70).

#### 3.1.2 Carboplatin

At the time of writing, direct involvement of lncRNAs in carboplatin resistance in CC has not been reported; however, several lncRNAs have been linked to carboplatin resistance in other gynecologic cancers. For example, deregulation of the IncRNA PVT1 has been deemed necessary for resistance in ovarian cancer (71). LncRNAs such as H19 and SNHG12, which are highly relevant in CC, have been associated with carboplatin resistance in ovarian cancer through the regulation of epigenetic mechanisms (72, 73).

#### 3.1.3 PD-1/PD-L1 inhibitors

Drugs targeted at programmed cell death protein-1 (PD-1) and programmed cell death ligand (PD-L1) are important immune checkpoint inhibitors (ICIs). PD-L1 is overexpressed in tumor cells or untransformed cells within the tumor microenvironment, inhibiting cytotoxic T cells by binding to the PD-1 receptor on activated T cells. PD-1 and PD-L1 inhibitors block this interaction, preventing cancer cells from evading the immune system and acting as ICIs by reactivating the T-cell-mediated tumor cell death (74). In CC, the specific therapy for PD-L1-positive is relatively new (55), so the resistance mechanism to this therapy is still to be studied. Some groups have reported a positive correlation between lncRNAs and PD-L1 expression. For example, MALAT1 is a ceRNA for miR-195 in lymphoma (75) and, in pancreatic cancer, LINC00473 sponges miR-195-5p (76); both increasing PD-L1 expression and both lncRNAs are overexpressed and are strongly associated with tumor development and poor prognosis in cervical cancer (The long noncoding RNA LINC00473, a target of microRNA 34a, promotes tumorigenesis by inhibiting ILF2 degradation in cervical cancer LncRNA MALAT1 Accelerates Cervical Carcinoma Proliferation by Suppressing miR-124 Expression in Cervical Tumor Cells). However, at the time of writing, no study correlates or characterizes any lncRNA and the development of resistance to PD-L1.

### 3.2 Second-line therapy

The second line of treatment comprises drugs such as Paclitaxel, Gemcitabine, Fluorouracil, and Irinotecan. These are used in patients whose progression continues even after first-line treatment and after 3–6 months of progression-free survival (55).

#### 3.2.1 Paclitaxel

LncRNAs are also associated with resistance to drugs used in combination with cisplatin in gynecologic cancers. The expression of the lncRNA KB-1471A8.2 can inhibit resistance to Paclitaxel in ovarian cancer, decreasing migration and invasion while increasing apoptosis. Overexpressing KB-1471A8.2 suppresses CDK4 expression, decreasing the proportion of cells in S phase and increasing the proportion in G0/G1 phase (77). On the other hand, lncRNA HEIH binds the EZH2 protein and participates in chromatin remodeling in hepatocellular carcinoma (78). It also enhances Paclitaxel tolerance in endometrial cancer through the activation of the Mitogen-activated protein kinase (MAPK) pathway proteins such as p38, C-Jun, and C-fos; although the consequential resistance mechanism is still unclear. (79). So, HEIH and KB-1471A8.2 are apparently antagonistic, but both lead to Paclitaxel resistance.

NEAT1 is overexpressed in CC, where it plays a key role in carcinogenesis and tumoral progression; this lncRNA also regulates cellular processes such as proliferation, migration, and invasion (25). NEAT1 has also been related to paclitaxel resistance in ovarian cancer, where it sponges miR-194, leading to ZEB1 overexpression. This protein is an Epithelial-Mesenchyme Transition (EMT)-inducing transcription factor. Thus, NEAT1 overexpression leads to enhanced tumor growth and low apoptosis levels in Paclitaxel-resistant ovarian cancer cells (80).

#### 3.2.2 Gemcitabine

LncRNA-mediated gemcitabine resistance mechanisms have been mostly described in pancreatic cancer through miRNA regulation. At least two lncRNAs promote proliferation, migration, invasion, and apoptosis evasion: HCP5 through the miR-214-3p/HDGF axis and it is also overexpressed in cervical cancer cell lines (81), and LINC00346 through the miR-188-3p/BRD4 axis It could represent an unexplored target in CC (82).

Elevated expression of AFAP1-AS1 in cervical cancer is associated with poor patient prognosis. Moreover, individuals in the gemcitabine-resistant group exhibited significantly higher levels of AFAP1-AS1 compared to those in the gemcitabine-sensitive group. Functionally, AFAP1-AS1 enhances tumor growth and contributes to gemcitabine resistance in cervical cancer cells. Mechanistically, AFAP1-AS1 regulates the expression of epidermal growth factor receptor (EGFR) by acting as a molecular sponge for miR-7-5p (83).

Notably, DLG1-AS1 expression was markedly elevated in gemcitabine-resistant HeLa/GEM and SiHa/GEM cervical cancer cells. Silencing DLG1-AS1 significantly reduced the viability and proliferation of these resistant cells. *In vivo*, tumors in nude mice with DLG1-AS1 knockdown showed reduced volume following gemcitabine treatment. DLG1-AS1 targets miR-16-5p, which regulates HDGF expression. Inhibition of miR-16-5p reversed the effects of DLG1-AS1 knockdown in gemcitabine-resistant cervical cancer cells (84). In fact, DLG1-AS1 has been identified as the most significantly upregulated lncRNA in cervical cancer tissues, and its elevated expression was associated with poor patient prognosis. Silencing DLG1-AS1 inhibited the proliferation of cervical cancer cells. Further analysis demonstrated that DLG1-AS1 acts as a competing endogenous RNA (ceRNA), binding to miR-107 and thereby preventing it from repressing its target gene, ZHX1 (85).

#### 3.2.3 Irinotecan

Little has been described regarding the involvement of lncRNAs in the processes leading to Irinotecan resistance. However, key lncRNAs have been detected, including CRNDE, H19, UCA1, HOTAIR, and MALAT1, which also play a key role in the development of CC. In addition, the resistance mediated by MALAT1 and HOTAIR (As mentioned above, both are important in CC) has been extensively linked to polymorphisms detected in both, enabling their use as potential biomarkers of irinotecan sensitivity in colorectal cancer (86, 87).

Clinically, elevated HOTAIR expression in cervical cancer tissues correlates with advanced tumor stage, lymph node metastasis, and decreased overall survival (88). Its potential as a prognostic biomarker is increasingly recognized, as it reflects tumor biological aggressiveness and clinical outcome (15). HOTAIR plays a crucial role in therapeutic resistance, since its overexpression has been linked to reduced sensitivity to both chemotherapy and radiotherapy (89). HOTAIR enhances DNA damage repair, inhibits apoptosis, and promotes survival signaling pathways, thereby diminishing the efficacy of cytotoxic treatments. Targeting HOTAIR or its downstream pathways may offer a promising strategy to overcome resistance and improve therapeutic responses in cervical cancer patients (reviewed in 90).

#### 3.2.4 5-Fluorouracile

We have already mentioned before the role of NEAT1 in Paclitaxel resistance, however, has also been reported as a precursor to generate cells resistant to other chemotherapy drugs. The study performed by Shao in 2021 showed the direct correlation between NEAT1 expression and 5-Fu) resistance in CC. the knocking down of NEAT1 by shRNA promoted the sensitivity of 5-Fu in CaSki cells due to the overexpression of miR-34a, this miRNA regulates the glycolysis through targeting LDHA, NEAT1 once again work as ceRNA to miR-34a inhibiting the interaction with its target mRNA and leading to 5-FU resistance in CC (91).

Substantially overexpressed in CC tissues, the lncRNA UCA1 can regulate proliferation, migration, and invasion by sponging miR-145 (92). Conversely, mir-23b-3p functions as a tumor suppressor, reducing the proliferation, migration, and invasion by targeting c-Met (93). The interaction between these ncRNAs has been characterized, albeit only in colorectal cancer. UCA1 sponges miR-23b-3p, leading to overexpression of the EMT-promoting ZNF281, which results in 5-Fu resistance (94). Another promising lncRNA is CCAT1, which promotes proliferation and invasion in cervical cancer but enhances 5-Fu sensitivity in colorectal cancer (95). This represents another instance of a resistance mechanism involving lncRNAs that are overexpressed in CC, which remain insufficiently characterized.

#### 3.2.5 Other second-line regimes

In addition to the drugs mentioned above, recent studies based on data from The Cancer Genome Atlas have revealed new drug targets. These molecules regulate signaling pathways that drive tumor progression. As a result, several drugs in clinical stages I-II have been identified as therapy candidates for CC, including therapies against VEGF (Bevacizumab), EGFR (Gefitinib), HER2 (Lapatinib), and tyrosine kinase receptors (Sunitinib) (96).

Despite the selectivity of targeted therapies, several lncRNAs have been reported to orchestrate resistance mechanisms to such drugs in various types of cancer. For example, the lncRNAs SNHG11 and CRART16 mediate resistance to bevacizumab through regulation of the miR-1207-5p/ABCC1 axis in colorectal cancer and miR-122-5p/FOS in gastric cancer, respectively (97, 98).

Gefitinib resistance has been associated with the expression of several lncRNAs, mainly in small cell lung cancer (NSCLC). The lncRNA MITA1, for example, favors an increase in cell viability and induction of autophagy in the presence of Gefitinib (99). Similarly, epigenetic regulations of lncRNAs such as UCA1, which is also overexpressed in CC, have also been associated with Gefitinib resistance through interaction with EZH2 (100–102). The lncRNA HOST2 favors Gefitinib resistance by sponging miR-621 (103), while CCAT1 allows resistance to this drug by repressing miR-218 in NSCLC (104), and the overexpression of these two lncRNAs promotes cell proliferation and migration (105, 106).

The extensive participation of lncRNAs in various biological processes has also allowed the recognition of lncRNAs as biomarkers of drug sensitivity or as indicators of resistance to therapies such as lapatinib. LncRNAs such as SPINT1-AS1 in the NCI-N87 and MCF7 cell lines and GAS5 in the SKBR-3/Tr cell line have been described as lapatinib sensitivity indicators (107). Overexpression of the lncRNA GIHCG has been also reported as a biomarker of lapatinib resistance (108).

LncRNAs also mediate resistance to sunitinib. HOTAIR regulates miR-17-5p, favoring autophagy in renal cancer (109); and the lncARSR/AXL/c-MET and lncRNECVSR/ERβ/Hif2 axes regulate cell proliferation, leading to sunitinib resistance in renal cell carcinoma (110, 111).

### 3.3 Signaling pathway inhibitors

On the other hand, there are reports of lncRNAs regulating signaling pathways such as ERK/MAPK, Wnt/β-catenin, and the PI3k/AKT as, have been related to lncRNAs, some of the most relevant that favor tumor development and drug resistance in CC (112). This has led to the development of drugs specifically targeting effectors of these signaling pathways. Within the ERK/MAPK pathway, relevance has been given to ERK inhibitors in BRAF or RAS mutations, highlighting ulixertinib, which is in the early clinical phases (113). In the PI3K/AKT pathway, several inhibitors targeting PI3K and mTOR proteins have been developed whose relevance has reached even clinical stages, including buparlisib (phase II clinical trial) and gedatosilb (phase Ib clinical trial) (114). Finally, for the Wnt/β-catenin pathway, the focus has been on the development of inhibitors targeting β-catenin, inhibitors of tankinase, and inhibitors of β-catenin/TCF interaction (CRT3/iCRT5/iCRT14/BC21/BC23/HI-B1), to mention a few (115).

Although the development of the signaling pathway inhibitors has advanced substantially, very little has been described about the resistance mechanisms themselves. Recent studies have allowed us to glimpse into new mechanisms by which lncRNAs favor resistance to specific inhibitors. HOTAIR, for example, has been highlighted as an important regulator of the Wnt/β-catenin and PI3K/AKT pathways even in the presence of the inhibitor ICRT14; knockdown of HOTAIR optimized inhibition and led to cell death in the HeLa cell line (40, 116).

## 4 Concluding remarks

**Figure 2:**
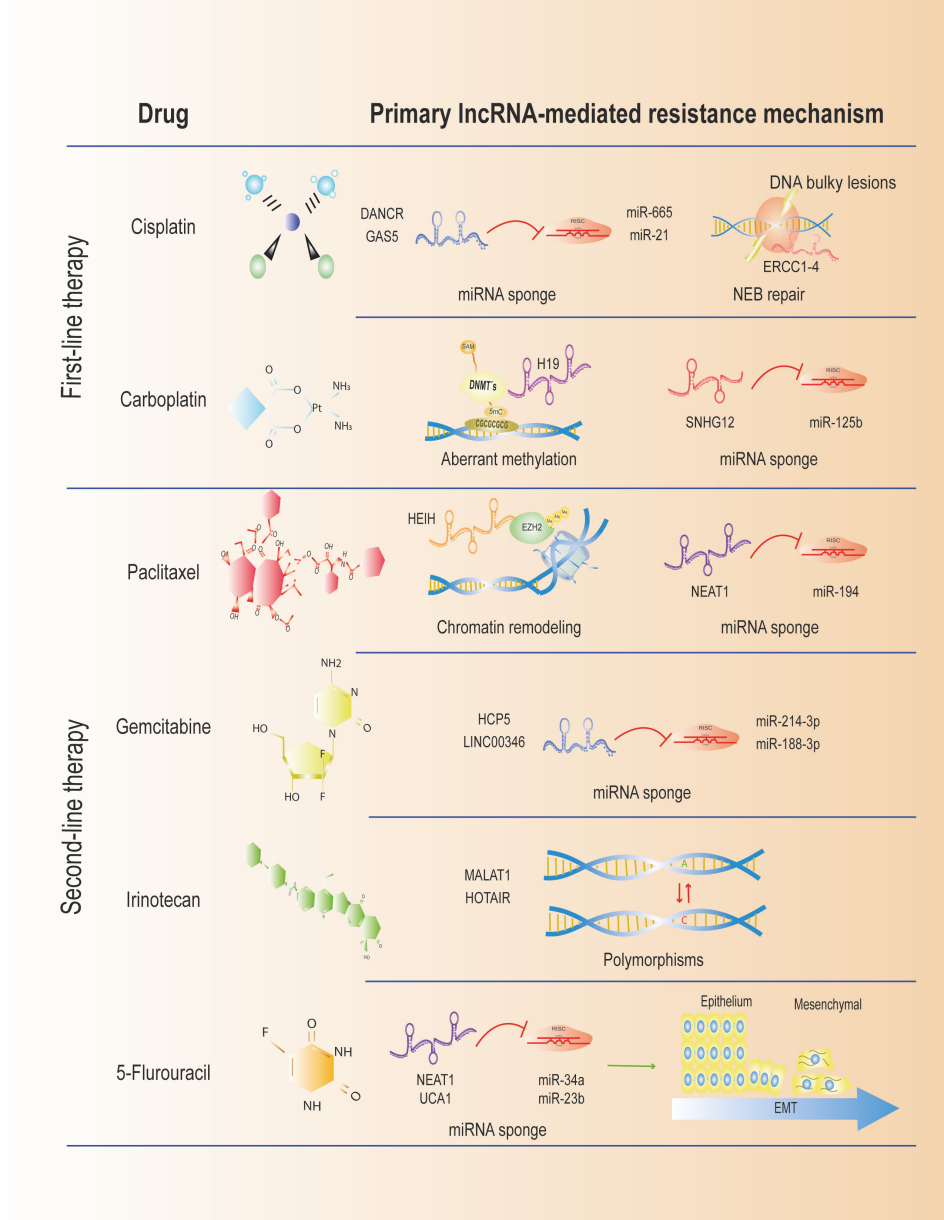
Treatment regimens for CC associated with LncRNAs. We show some described lncRNAs mediating resistance in the first- and second-line therapies.

After reviewing the available literature on lncRNAs and drug resistance in CC, it is evident that only a few lncRNAs are directly associated with chemoresistance to some therapies. The most widely described mechanism is the competitive binding of lncRNAs to miRNAs, which prevents the downregulation of their mRNA targets. This ceRNA effect results in the alteration of cellular processes, such as EMT, migration, invasion, and proliferation, ultimately leading to chemoresistance to standard treatments. However, when compared to other gynecological cancers, such as ovarian and endometrial, in which the same drugs are employed, cervical cancer remains poorly explored (**Figure 2**). Interestingly, the expression profiles of lncRNAs that promote chemoresistance are similar in CC and other gynecological cancers, providing insight into the mechanisms that may underlie the development of chemoresistance to therapies used in CC. The study of lncRNAs and the drug resistance mechanisms they participate in is a promising tool for developing better therapeutic schemes that will undoubtedly expand in the following years, resulting in more favorable clinical outcomes for patients with CC.
